# Ultra-rare severe kidney dysplasia mimicking salt-wasting tubulopathy associated with *TFCP2L1* gene variants

**DOI:** 10.1007/s00467-025-06804-3

**Published:** 2025-06-26

**Authors:** Manuel Vaqueiro Graña, Leire Madariaga, Sara Gómez-Conde, Ainhoa Iceta Lizarraga, Josune Hualde Olascoaga, Gema Ariceta

**Affiliations:** 1 Department of Pediatric Nephrology, Vall D’Hebron University Hospital, Barcelona, Spain; 2 Pediatric Nephrology Department, CIBERDEM/CIBERER/Endo-ERN, Biobizkaia Health Research Institute, University of the Basque Country, Cruces University Hospital, Plaza de Cruces S/N, 48903 Barakaldo, Spain; 3 Pediatric Department, University of the Basque Country, Biobizkaia Health Research Institute, CIBERDEM/CIBERER/Endo-ERN, Barakaldo, Spain; 4 Department of Pediatric Nephrology, Navarra University, Hospital, Pamplona/Iruña, Spain; 5 Autonomous University of Barcelona, Barcelona, Spain

**Keywords:** Phenocopy, Salt-wasting tubulopathy, Chronic kidney disease, Monogenic

## Abstract

Rare monogenic diseases are increasingly identified in children with chronic kidney disease. We describe a consanguineous preterm male infant with a clinical picture of advanced kidney dysfunction and severe renal salt-wasting, highly suggestive of prenatal onset Bartter syndrome. Patient’s follow-up was characterized by severe polyuria; episodes of hyponatremia, hypokalemia, and hypochloremia; and metabolic alkalosis and hyperuricemia. We found a homozygous pathogenic variant in the *TFCP2L1 *gene, a transcription factor required for normal kidney development, that regulates acid–base and salt–water homeostasis. To our knowledge, there is only one published case of a child with *TFCP2L1 *gene pathogenic variants with a similar phenotype. This report adds evidence to *TFCP2L1* as a cause of monogenic kidney disorders. Rare kidney dysplasias may manifest as phenocopies of primary tubulopathies. Genetic diagnosis plays a major role and should be carefully considered in patients with refractory course to standard treatment to facilitate management and family counselling.

## Introduction

Monogenic diseases are a well-established cause of chronic kidney disease (CKD) in children, where genetics play a key role in improving diagnosis and future management. Nevertheless, some inherited kidney disorders can mimic other conditions. This paper aims to describe an infant with an ultra-rare monogenic kidney dysplasia secondary to likely pathogenic homozygous *TFCP2L1* variants, who presented with severe polyhydramnios and polyuria and massive renal salt-wasting since birth resembling prenatal onset Bartter syndrome.

## Case report

An eight-month-old male infant was referred to our center with the clinical diagnosis of prenatal onset Bartter syndrome, severe polyuria, advanced CKD, and poor response to treatment with sodium chloride and potassium chloride supplements, associated with ibuprofen first or indomethacin later. Initial genetic testing for *SLC12A1*, *KCNJ1*, *BSND*, *CLCNKA*, *CLCNKB*, *MAGED2*, and *SLC12A3* genes was negative. Further diagnostic work-up including a panel of additional genes associated with hypokalemic metabolic alkalosis (*CASR*, *KCNJ10*, *SCNN1B*, *SCNN1G*, *SCN4A*, *CLCN5*, *OCRL1*, *HNF1B*, *SCNN1A*, *NR3C2*) turned out to be also negative.

He was the 2nd child from consanguineous parents, without a record of family kidney diseases, born after cesarean delivery at 34 weeks of gestation complicated by polyhydramnios since the 29th week. He was born with normal weight and length for age (Table 1), and normal physical exam except significant hypotonia. After birth, he presented early and severe polyuria (7 mL/kg/day), associated with severe metabolic alkalosis, hyponatremia, and hypochloremia of renal origin, but normocalciuria. Other findings included remarkable hyperuricemia, mild hypercalcemia (2.84 mmol/L), and hypermagnesemia (1.28 mmol/L) associated with severe kidney dysfunction (Table 1). Initially, kidney ultrasound was reported as normal. No extrarenal manifestations were observed. He was treated with high fluids and electrolyte supplementation (up to 28.4 mmoL/kg/day of NaCl and up to 196 ml/kg/day of fluids), and transiently discharged home at 1.5 months of life with enteral feeding. He continued with polyuria and suffered from several episodes of hyponatremic, hypochloremic, and hypokalemic severe metabolic alkalosis, and progressive CKD (Table 1). Repeated ultrasound at 4 months of age showed that both kidneys were small, at the lower normal range for age, with diffuse cortical echogenicity lack of corticomedullary differentiation, without nephrocalcinosis. Patient management during the first months of life became extremely difficult due to treatment with high doses of electrolyte supplementation, feeding difficulties, prolonged hospitalization, and delayed growth and development. Prostaglandin inhibition with ibuprofen or indomethacin was ineffective and worsened patient eGFR.

**Table 1:** Growth and laboratory findings during patient follow-up and treatment with NaCl supplementation

	At birth	24 h of life	1 month	8 months	12 months	16 months	24 months
Weight (kg) (percentile, SD)	2.29 (70, 0.55)	-	2.785 (< 1, − 2.73)	6.12 (< 1, − 2.49)	8.18 (8, − 1.39)	10.04 (15, − 1.07)	11.840 (18,−0.94)
Length cm (percentile, SD)	46 (81, 0.89)	46 (81, 0.89)-	49.5 (1, − 2.11)	64 (< 1, − 2.76)	71.3 (4, − 1.75)	75.5 (5, − 1.75)	85.5 (10, − 1.32)
Head circumference cm (percentile, SD)	31 (50, 0)	31 (50, 0)					46.5 (< 1, − 2.41)
Blood pressure (mmHg)	77/43	-	74/35	90/50	104/62	114/73	103/69
Blood							
Serum creatinine (mg/dL)	0.63	1.45	0.79	1.97	1.26	0.94	1.09
Serum cystatin C (mg/L)	-	-	3.85	8.69	4.4	3.95	3.33
eGFR (Schwartz 2009) mL/min/1.73 m^2^	30.1	13.1	25.9	13.4	23.3	33.1	32.4
eGFR (creatinine-cystatin C-based CKiD) mL/min/1.73 m^2^			21	11	20	24	27
Serum Na (mmol/L)	139	121	136	138	141	142	136
Serum K (mmol/L)	4.8	4.6	5.1	3.79	3.71	3.79	3.5
Serum Cl (mmol/L)	109	80	92	91	98	97	99
Serum uric acid (mg/dL)	-	-	16.1	12.4	11.3	7.3	10.5
Acid–base balance (venous)							
pH	7.35	7.51	7.49	7.68	7.47	7.47	7.46
CO2	46.8	28.9	42.6	33	43	51	42
HCO3	25.6	22.7	31.7	39	31.3	37.1	29.9
Urine							
K (mmol/L)/FEK	-	16/84%	-	12.9/51.6%	7.9/29.8%	4.9/15.1%	3.2/9.06%
Prostaglandin E (pg/mL) (normal range up to 1850)	-	-	-	723	-	-	-
Plasma renin activity ng/mL/h (normal range: 1.4–7.8)	-	-	-	0.8	-	-	-
Plasma aldosterone level ng/dL (normal range: 6.1–43.71)	-	-	-	59.1	-	-	-

*eGFR* estimated glomerular filtration rate (*not validated for children younger than 1-year-old) expressed in mL/min/1.73 m^2^, *FEK* fractional excretion of potassium, *SD* standard deviation

Upon arriving to our center at 8 months, indomethacin was discontinued due to advanced CKD. Enteral feeding was started through a nasogastric tube first and gastro-jejunostomy later. The child had increased serum aldosterone levels and low plasma renin activity. Urinary prostaglandin E and complementary exams (echocardiogram, funduscopy, esophagogram, central nervous system magnetic resonance, and hormonal studies) were normal (Table 1). A new ultrasound revealed normal-sized but dedifferentiated kidneys without nephrocalcinosis.

Over time, we achieved better patient control and development after intensive nutrition, water, and electrolyte supplementation, which enabled discharge from hospital. Currently, he is 2 years old, with normal weight and growth. He has a severe renal salt-wasting phenotype associated with CKD 4 (eGFR Schwartz 2009 32.4 mL/min/1.73 m^2^; eGFR creatinine-cystatin C-based CKiD 27 mL/min/1.73 m^2^). He developed hypertension at 12 months of age and was treated with two drugs (carvedilol and amlodipine). He still receives salt supplementation (15 mmoL/kg/day) to maintain plasma electrolytic balance. Tubular proteinuria has been noted (β2-microglobulin 1.61 mg/L (normal value: 0–0.10); β2-microglobulin/creatinine ratio of 20,125 µg/g) with no albuminuria.

Additional genetic analyses with whole exome sequencing demonstrated a homozygous variant in the *TFCP2L1* gene (*TFCP2L1* (NM_014553.3):c.1279 del; p.(Gln427Serfs*18)) encoding the transcription factor CP2-like 1. This variant is predicted to lead to a frameshift transcript, resulting in either a truncated protein or non-sense mediated mRNA decay. This variant has not been reported previously, either in ClinVar or in Human Gene Mutation database (HGMD®) and it is not present in the Genome Aggregation Database (gnomAD). Currently, this variant is classified as “likely pathogenic” according to the American College of Medical Genetics (ACMG-AMP) guidelines (PVS1, PM2) [1]. However, the evidence of this gene as a monogenic cause of kidney dysplasia and salt-losing tubulopathy is limited due to the presence of only one patient with a similar clinical picture previously reported [2]. Segregation analysis showed heterozygous carrier status of the parents.

## Discussion

In this report, we present the case of a child with early kidney failure presumably associated with a rare monogenic kidney dysplasia with severe salt-losing tubulopathy. Previously only one child with a comparable clinical picture and a similar homozygous variant in the same gene (*TFCP2L1* – c.869 delC; p.(Ser290Phefs*50)) has been reported. He suffered severe metabolic alkalosis with hyponatremia, hypochloremia, and hypokalemia, and developed hypertension and kidney failure requiring hemodialysis at 3.5 years of age. Unlike our case, he had associated congenital cataracts and seizures [2].

Previous studies in mice have demonstrated that CP2-like 1 is a transcription factor expressed throughout kidney development and it is essential for the maturation of the distal nephron. It facilitates the establishment and maintenance of pluripotency in embryonic stem cells. Specifically, the *TFCP2L1* gene is required for normal duct development in the salivary gland and kidney. It is also expressed in other exocrine glands. It coordinates the development of the kidney collecting ducts, including the intercalated and principal cells, which regulate acid–base and salt–water homeostasis, respectively. Further, a knock-out mice model for the *TFCP2L1* gene exhibits hypoplastic kidneys at birth. However, the potential developmental mechanisms leading to kidney dysplasia in those cases have not been extensively studied [2–4].

Interestingly, knockout *TFCP2L1* mice showed reduced expression of distal nephron markers, possibly explaining the mild hypercalcemia and hypermagnesemia following increased sodium reabsorption in the thick ascending limb of the loop of Henle [2–4]. Differential diagnosis with Bartter syndrome of antenatal origin can be challenging due to a similar clinical picture [5], and comparable serum and urine electrolyte composition, resulting from severely impaired tubular sodium handling. Nevertheless, our patient did not exhibit hypercalciuria nor nephrocalcinosis, as expected in severe Bartter syndrome. In contrast to the previously reported patient, our case had elevated uric acid levels, which could be explained by a compensatory mechanism of enhanced reabsorption in the proximal tubule due to volume contraction and salt-wasting nephropathy. In addition, progressive CKD could have also contributed to the hyperuricemia. The presence of hypertension in this child with presumed salt-wasting was unexpected and could be related to progressive CKD and impaired volume regulation. However, the hypertension in this patient could also reflect an excessive salt supplementation, which would explain the suppressed renin level.

In conclusion, we have described an infant with a homozygous likely pathogenic variant of the *TFCP2L1* gene who presented CKD, bilateral kidney dysplasia, and impaired distal nephron function. Our case report adds new knowledge and provides further evidence that alterations in the *TFCP2L1* gene should be considered as a potential cause in patients with CKD and severe renal salt-wasting.

## Summary

### What is new

- *TFCP2L1* variants may cause kidney dysplasia and impaired distal nephron development, leading to a mixed phenotype of CKD and mimicking salt-wasting tubulopathy. Genetic analysis is essential for management and counselling.

## Data Availability

The data presented in this study are from a single individual patient. These data are not publicly available to protect patient confidentiality and comply with ethical standards.
